# Influence of fat-based versus glucose-based enteral nutrition formulas on glucose homeostasis

**DOI:** 10.1186/cc12188

**Published:** 2013-03-19

**Authors:** M Wewalka, A Drolz, C Zauner

**Affiliations:** 1 Medical University of Vienna, Austria

## Introduction

Trauma patients who receive fat-based parenteral nutrition (PEN) achieve better glucose control compared with those fed with glucose-based PEN [1,2]. Therefore, we determined whether fat-based enteral nutrition (EN) has the same benefit on glucose control and exogenous insulin demand in medical intensive care patients compared with glucose-based EN. Here we present preliminary data for this ongoing randomized controlled cohort study.

## Methods

Medical critically ill patients with need for mechanical ventilation and without contraindications for EN are included in the study. Patients are randomly assigned to receive either fat-based (*n = *30) or glucose-based (*n = *30) EN. To evaluate the individual calorie demand, indirect calorimetry is performed after an overnight fast. The determined amount of EN is administered continuously for 5 days. Glucose concentrations are measured at least three times per day and averaged. Furthermore, exogenous insulin demand per 24 hours and calorie achievement per 24 hours are evaluated daily.

## Results

So far, 37 patients, 16 with fat-based, 21 with glucose-based EN have been included in the study. Both groups had similar age (62 ± 10 vs. 58 ± 16 years, *P *= 0.44), body mass index (26.7 ± 5.8 vs. 28.4 ± 4.4 kg/ m^2^, *P *= 0.302), SAPS II score (62.4 ± 12.7 vs. 64 ± 12.3, *P *= 0.697), and fasting plasma glucose (132 ± 34 vs. 121 ± 26 mg/dl, *P *= 0.269). Furthermore, resting energy expenditure was similar in both groups (1,522 ± 365 vs. 1,573 ± 313 kcal/day, *P *= 0.647). Throughout the entire study period, average blood glucose, exogenous insulin demand, and calorie achievements per day were similar between the groups (day 1: gluc: 139 ± 30 vs. 127 ± 20 mg/dl, *P *= 0.143; ins: 27.8 ± 28.4 vs. 16.2 ± 19.4 IE, *P *= 0.155; EN: 52.9 ± 25.6 vs. 61.7 ± 25%, *P *= 0.304; day 3: gluc: 129 ± 17 vs. 133 ± 21 mg/dl, *P *= 0.578; ins: 35.1 ± 30.9 vs. 32.3 ± 30.5 IE, *P *= 0.802; EN: 78.3 ± 28.9 vs. 83.4 ± 29%, *P *= 0.617; day 5: gluc: 124 ± 16 vs. 132 ± 17 mg/dl, *P *= 0.276; ins: 25.7 ± 37 vs. 33 ± 26.4 IE, *P *= 0.562; EN: 73.9 ± 41.4 vs. 87.1 ± 23%, *P *= 0.354). Interestingly, the calorie achievement was not associated with insulin demand (day 1: *R *= 0.241, *P *= 0.156) or average blood glucose (day 1: *R *= 0.248, *P *= 0.14) throughout the study.

## Conclusion

Medical critically ill patients with fat-based or glucose- based EN achieve similar glucose control. EN was not associated with glucose concentrations or insulin demand.
